# *In silico* analysis of the solute carrier (SLC) family in cancer indicates a link among DNA methylation, metabolic adaptation, drug response, and immune reactivity

**DOI:** 10.3389/fphar.2023.1191262

**Published:** 2023-06-15

**Authors:** Alessandro Lavoro, Luca Falzone, Barbara Tomasello, Giuseppe Nicolò Conti, Massimo Libra, Saverio Candido

**Affiliations:** ^1^ Department of Biomedical and Biotechnological Sciences, University of Catania, Catania, Italy; ^2^ Epidemiology Unit, IRCCS Istituto Nazionale Tumori “Fondazione G. Pascale”, Naples, Italy; ^3^ Department of Drug and Health Sciences, University of Catania, Catania, Italy; ^4^ Research Center for Prevention, Diagnosis and Treatment of Cancer, University of Catania, Catania, Italy

**Keywords:** cancer, TCGA, DNA methylation, epigenetics, solute carrier, SLCs, drug resistance, bioinformatics

## Abstract

**Introduction:** The oncogenic transformation is driven by genetic and epigenetic alterations influencing cancer cell fate. These alterations also result in metabolic reprogramming by modulating the expression of membrane Solute Carrier (SLC) transporters involved in biomolecules trafficking. SLCs act as tumor suppressors or promoters influencing cancer methylome, tumor growth, immune-escape, and chemoresistance.

**Methods:** This *in silico* study aimed to identify the deregulated SLCs in various tumor types compared to normal tissues by analyzing the TCGA Target GTEx dataset. Furthermore, the relationship between SLCs expression and the most relevant tumor features was tackled along with their genetic regulation mediated by DNA methylation.

**Results:** We identified 62 differentially expressed SLCs, including the downregulated *SLC25A27* and *SLC17A7*, as well as the upregulated *SLC27A2* and *SLC12A8*. Notably, *SLC4A4* and *SLC7A11* expression was associated with favorable and unfavorable outcome, respectively. Moreover, *SLC6A14, SLC34A2,* and *SLC1A2* were linked to tumor immune responsiveness. Interestingly, *SLC24A5* and *SLC45A2* positively correlated with anti-MEK and anti-RAF sensitivity. The expression of relevant SLCs was correlated with hypo- and hyper-methylation of promoter and body region, showing an established DNA methylation pattern. Noteworthy, the positive association of cg06690548 (*SLC7A11*) methylation with cancer outcome suggests the independent predictive role of DNA methylation at a single nucleotide resolution.

**Discussion:** Although our *in silico* overview revealed a wide heterogeneity depending on different SLCs functions and tumor types, we identified key SLCs and pointed out the role of DNA methylation as regulatory mechanism of their expression. Overall, these findings deserve further studies to identify novel cancer biomarkers and promising therapeutic targets.

## 1 Introduction

The multistep process of malignant transformation is driven by some genetic and epigenetic alterations leading to the aberrant function of key genes involved in the growth, survival, and antitumoral surveillance of normal cells. In the last few years, several studies have highlighted the involvement of DNA methylation in genetic reprogramming occurring during cancer development (Nishiyama and Nakanishi, 2021). DNA methylation is catalyzed by DNA methyltransferases (DNMTs), which transfer a methyl group from the donor S-adenosyl-L-methionine (SAM) to CpG dinucleotides of the DNA sequence (Greenberg and Bourc’his, 2019). Aberrant biosynthesis of SAM and the deregulation of the DNMTs contribute to shape cancer methylome leading to inappropriate expression or silencing of several genes associated with several cancer processes (Serefidou et al., 2019; Erichsen et al., 2022). Among these, the adaptation of metabolic processes is a milestone of transforming cells required to support the constant requirement of biosynthetic and energetic supplies for cell growth and proliferation (Hanahan and Weinberg, 2011). This metabolic adaptation is supported by aberrant expression of membrane transport proteins belonging to the Solute Carrier (SLC) family responsible for the efflux or influx of numerous inorganic ions and biomolecules. The trafficking of these molecules (i.e., ions, glucose, amino acids (AAs), lactate, and lipids) is mediated by either passive facilitative or secondary active transport mechanisms (Zhang et al., 2019). The SLC transporters comprise over 450 proteins organized into more than 60 families based on sequence similarity and functional annotation. The SLC proteins are ubiquitously distributed and mostly localized in the cell membrane, mitochondria, and other intracellular organelles including melanosomes (Pizzagalli et al., 2021).

Over the years, a growing number of studies have highlighted the involvement of SLCs in cancer development with tumor suppressor or promotive functions depending on cancer type. For instance, tumor-promoting SLCs have been described to be involved in angiogenesis, metastatic growth, immune escape, drug resistance, and Epithelial-Mesenchymal Transition (EMT). Conversely, the downregulation of several SLCs promotes tumor growth and proliferation, suggesting their role as tumor suppressor genes (Rashid et al., 2021). Notably, several SLCs, including the organic anion or cation, nucleosides, carnitine, and copper transporters, are directly involved in either chemoresistance or chemo sensitivity mediating influx and efflux of anticancer drugs and affecting their pharmacokinetics, efficacy, and side effects (Roth et al., 2012; Zhou et al., 2017; Brecht et al., 2020). Concomitantly, the dysregulation of some SLCs can contribute to drug resistance altering various mechanisms involved in apoptosis, stemness, detoxification, and drug-induced DNA damage repair (Gottesman et al., 2002; Huang and Sadée, 2006; Li and Shu, 2014).

Moreover, many other SLCs support the metabolism of cancer cells, which is characterized by high consumption of glucose and subsequent lactate production even in the presence of oxygen, known as aerobic glycolysis or Warburg effect (Kocianova et al., 2022). Extensive literature has also reported that the reprogramming of lactate metabolism related to SLCs occurs in various cellular components of the Tumor Microenvironment (TME), such as endothelial cells, cancer stem cells, cancer-associated fibroblasts, and immune cells. Notably, tumor cell sustainment is also guaranteed by the TME cells that release lactate (Fischer et al., 2007; Sonveaux et al., 2012; Rashid et al., 2021). In this context, the main oncometabolism hallmarks include the upregulation of glucose (*SLC2A* family) and lactate transporters (*SLC16A* family), cystines (*SLC7A11*), glutamine (*SLC1A5*), and leucine (*SLC7A5*/*SLC3A2*) transporters, which provide the substrates for lactate dehydrogenase and enzymes of glycolytic and pentose-phosphate pathways (Ancey et al., 2018; Kozal et al., 2021). These metabolic changes also enhance the synthesis of nucleotides, lipids, and proteins, the Tricarboxylic Acid cycle anaplerosis, as well as the glutathione synthesis for redox homeostasis and maintenance of intracellular pH (De Berardinis et al., 2007; Felmlee et al., 2020; Liu et al., 2020).

Glucose metabolic reprogramming is also involved in cancer immunological response through the alteration of SLCs expression patterns in the immune cells (Chen and Chen, 2022). For instance, T cell activation is regulated by alanine transporter *SLC38A1* (Ron-Harel et al., 2019), the macrophage polarization is also mediated by *SLC6A8*-dependent creatine uptake (Ji et al., 2019), and the redox homeostasis and antigen presentation of Dendritic cells are promoted by *SLC3A2* and *SLC7A11* (D'Angelo et al., 2010; Brombacher and Everts, 2020). Furthermore, some *SLC16*s alter the anti-tumor functions of tumor-infiltrating lymphocytes (O'Sullivan et al., 2019) including the T reg cells, which maintain their suppressive activity by *SLC16A1* overexpression involved in the lactate uptake (Watson et al., 2021).

In addition, the altered glucose metabolism also promotes the one-carbon metabolism responsible for SAM biosynthesis, which in turn affects the cancer methylome (Locasale, 2013; Rashid et al., 2021). Therefore, close crosstalk between metabolic alteration and DNA methylation is responsible for the aberrant expression of some SLCs revealing attractive molecular feedback. However, there is a lack of information on the comprehensive gene regulation mechanisms of SLC transporters in cancer, including the role of DNA methylation.

On these bases, we performed an *in silico* evaluation of the expression of SLC family members and the involvement of DNA methylation in their regulation in different cancer types. To this purpose, we used the gene expression and DNA methylation data available in the TCGA Target GTEx and TCGA Pan-Cancer datasets, respectively. A detailed overview is provided on the main SLCs involved in the transport and homeostasis of several molecules essential for tumor survival and proliferation. Moreover, SLCs involvement in the tumor immune microenvironment was investigated by analyzing the association between their expression and the intratumoral immune signatures. Finally, the relationship between pharmacological response and the expression of SLCs was examined to identify some SLCs that confer drug sensitivity or resistance to tumor cells.

## 2 Materials and methods

### 2.1 Data collection from public repositories datasets

In this study, the TCGA TARGET GTEx (*N* = 10,534 TCGA tumor and *N* = 7,791 GTEx normal samples), the TCGA Pan-Cancer (*N* = 10,535 RNAseq gene expression and *N* = 9,639 Infinium 450K DNA methylation samples), and Cancer Cell Line Encyclopedia (CCLE) cohorts (N = 1.076 RNAseq gene expression samples and *N* = 504 pharmacologic profiles for 24 anticancer drugs across 504 CCLE lines) were used to analyze the gene expression and DNA methylation status of 429 SLC genes (Supplementary Table S1). The list of SLC families and information on their members were achieved from BioParadigms (https://www.bioparadigms.org/slc/intro.htm—accessed on 27 May 2022). In this study, all SLC gene identities used in each Dataset were uniformed according to the “SLC Name” reported in Supplementary Table S1. UCSC Xena Functional Genomics Explorer (https://xenabrowser.net/, accessed on September 2022) was used to retrieve gene expression and DNA methylation data of selected SLC genes to perform Pearson’s correlation and DNA methylation status analyses.

The abbreviation and extended full name of the 33 cancer types are reported in Table 1.

**TABLE 1:** Abbreviations and full names of TCGA cancer types.

**Abbreviation**	**TCGA pan-cancer - tumor type**	**TCGA TARGET GTEx**
ACC	Adrenocortical Cancer	Adrenocortical Cancer
BLCA	Bladder Urothelial Carcinoma	Bladder Cancer
BRCA	Breast Invasive Carcinoma	Breast Cancer
CESC	Cervical & Endocervical Cancer	Cervical Cancer
CHOL	Cholangiocarcinoma	Bile Duct Cancer
COAD	Colon Adenocarcinoma	Colon Cancer
DLBC	Diffuse Large B-Cell Lymphoma	Large B-cell Lymphoma
ESCA	Esophageal Carcinoma	Esophageal Cancer
GBM	Glioblastoma Multiforme	Glioblastoma
HNSC	Head & Neck Squamous Cell Carcinoma	Head and Neck Cancer
KICH	Kidney Chromophobe	Kidney Chromophobe
KIRC	Kidney Clear Cell Carcinoma	Kidney Clear Cell Carcinoma
KIRP	Kidney Papillary Cell Carcinoma	Kidney Papillary Cell Carcinoma
LAML	Acute Myeloid Leukemia	Acute Myeloid Leukemia
LGG	Brain Lower Grade Glioma	Lower Grade Glioma
LIHC	Liver Hepatocellular Carcinoma	Liver Cancer
LUAD	Lung Adenocarcinom	Lung Adenocarcinoma
LUSC	Lung Squamous Cell Carcinom	Lung Squamous Cell Carcinoma
MESO	Mesotheliom	Mesothelioma
OV	Ovarian Serous Cystadenocarcinoma	Ovarian Cancer
PAAD	Pancreatic Adenocarcinoma	Pancreatic Cancer
PCPG	Pheochromocytoma & Paraganglioma	Pheochromocytoma & Paraganglioma
PRAD	Prostate Adenocarcinoma	Prostate Cancer
READ	Rectum Adenocarcinoma	Rectal Cancer
SARC	Sarcoma	Sarcoma
SKCM	Skin Cutaneous Melanoma	Melanoma
STAD	Stomach Adenocarcinoma	Stomach Cancer
TGCT	Testicular Germ Cell Tumor	Testicular Cancer
THCA	Thyroid Carcinoma	Thyroid Cancer
THYM	Thymoma	Thymoma
UCEC	Uterine Corpus Endometrioid Carcinoma	Endometrioid Cancer
UCS	Uterine Carcinosarcoma	Uterine Carcinosarcoma
UVM	Uveal Melanoma	Ocular melanomas

### 2.2 Differential analyses of SLCs gene expression

Differential analysis between each TCGA tumor type and GTEx normal tissues, pooled all together as one control group, was performed to identify the SLC genes differentially expressed in cancer. This approach, based on the same global pool of GTEx tissues, allowed us to compare SLC Differentially Expressed Genes (DEGs) among all TCGA tumor types. Since the Xena Differential Gene Expression Analysis tool can analyze up to 2000 samples, the differential analysis was performed using R software version 4.2.0 (https://www.r-project.org/) to compute the log_2_FC as the difference between the log_2_ expression means of each tumor type and the pooled GTEx control group, whereas the *p*-value was calculated using two-tailed Student’s t-Test (see Supplementary Materials for R code and Raw data 1). In addition, to avoid the underestimation of SLC DEGs in specific tissue environment, we carried out differential analysis between each TCGA tumor type and matched normal tissue (Table 1) using the Xena Differential Gene Expression Analysis tool (https://xenabrowser.net/) on TCGA TARGET GTEx cohort.

Furthermore, the Xena Differential Gene Expression Analysis tool was also used to identify the SLC genes differentially expressed between the comparison groups in TCGA Pan-Cancer cohort. In particular, the patients were divided into two groups for each tumor type according to Overall Survival (OS) and Progression Free Interval (PFI) at 5 years separately, as well as the intratumoral immune state C2-C3 (immune-response) *versus* C4-C6 (immuno-quiet) and analyzed using XENA UCSC Limma-Voom pipeline. The immune-response and immune-quiet signatures were obtained from “Subtype_Immune_Model_Based” in analytic data type available on Xena UCSC (Thorsson et al., 2018).

The difference between each comparison group was expressed as log_2_FC, whereas the *p*-value ≤0.05 was considered statistically significant.

### 2.3 Correlation analysis between gene expression and DNA methylation

Pearson’s correlation analyses between gene expression RNAseq (Batch effects normalized mRNA data) and DNA methylation (Methylation 450 K) levels of selected SLC genes retrieved from the TCGA Target GTEx cohort were performed using cor_test function of rstatix package executed on R software version 4.2.0 (https://www.r-project.org/) (see Supplementary Materials for R code and Raw data 2).

### 2.4 OS and PFI analyses according to CG probeset methylation levels

Kaplan Meier survival analysis was performed using GraphPad Prism 8 (version 8.0.2) (GraphPad Software, San Diego, CA, United States). Log-rank (Mantel-Cox) test was used to calculate the *p*-value and median survival times of each comparison group. For each TCGA Pan-Cancer tumor type, the patients were divided into Up (upper the median DNA methylation level) and Down (lower median DNA methylation level) methylation groups according to the methylation levels of the cg06690548 (*SLC7A11*).

### 2.5 Data visualization

Heatmap of SLC DEGs and clustering analyses were performed using Heatmapper (http://heatmapper.ca). Volcano plot was implemented using the publicly available tool VolcaNoseR (https://huygens.science.uva.nl/VolcaNoseR2/). Alluvial plot was drawn using the RAWGraphs tool (https://www.rawgraphs.io/). Correlation and DNA methylation graphs were adapted from GraphPad Prism (version 8.0.2) (GraphPad Software, San Diego, CA, United States) scatter dot plots in which each dot indicates a Pearson’s correlation value or median CG probeset DNA methylation levels computed for each tumor type.

## 3 Results

### 3.1 Gene expression analysis of SLC family genes by TCGA tumor type

**FIGURE 1:**
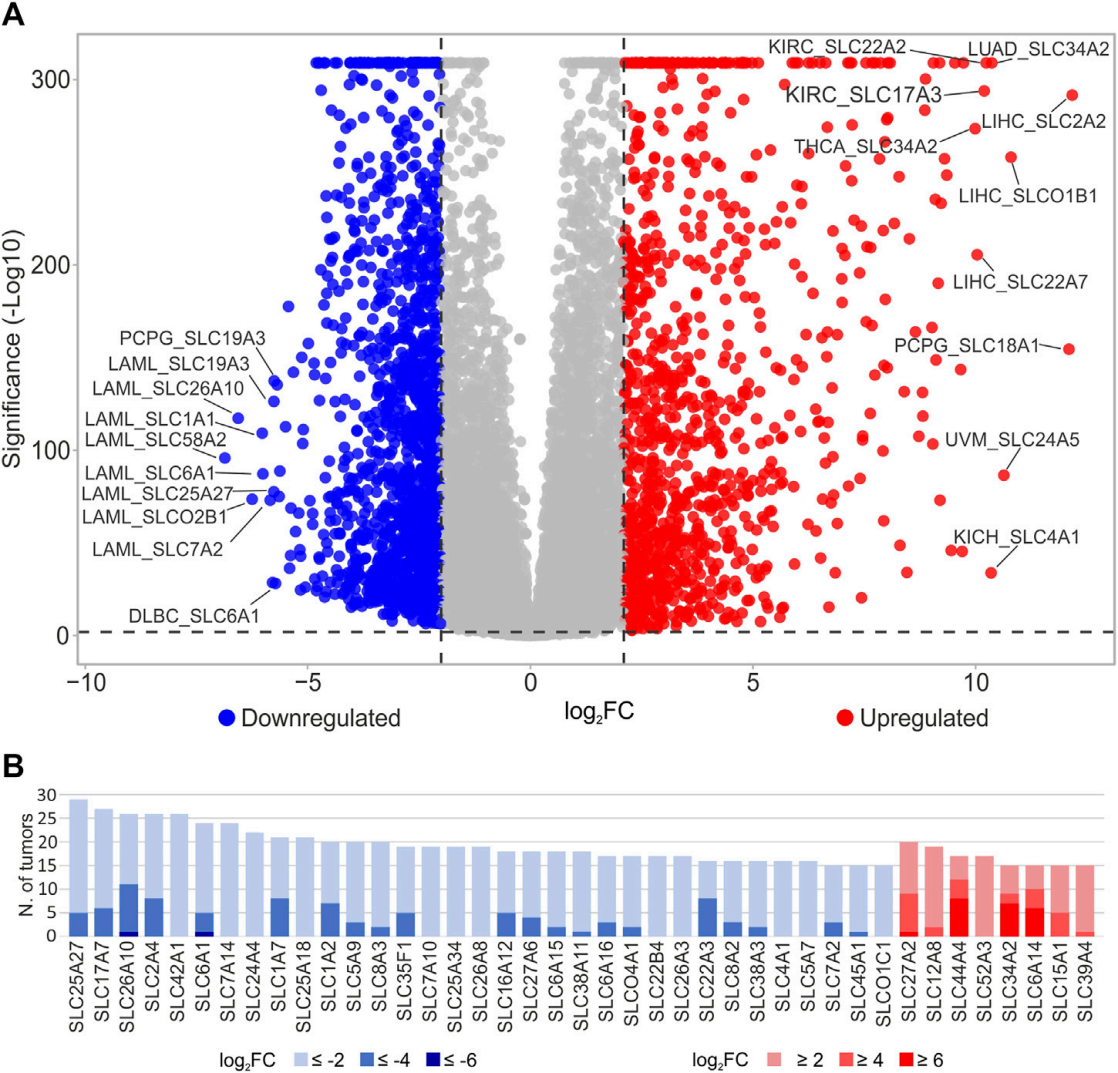
Differential analysis of SLC genes in TCGA tumor types. **(A)** DEGs SLC reported in the Volcano plot analysis were obtained comparing the expression of SLC genes between each TCGA tumor type and pooled GTEx control group. Blue dots indicate the downregulated SLCs with log2FC ≤ −2 (*p* ≤ 0.05), whereas red dots represent the upregulated genes log2FC ≥ 2; *p* ≤ 0.05). Top ten down- and upregulated SLC genes and relative tumor type were labeled. The *p*-values were represented as -Log10. **(B)** Distribution of significantly (*p* ≤ 0.05) up- (red) and downregulated (blue) SLC genes in at least 15 tumors with respect to pooled GTEx control group according to different log_2_FC values cutoff (≤-2 or ≥2; ≤ −4 or ≥4; ≤ −6 or ≥6).

To evaluate the involvement of the SLC genes (*N* = 429, Supplementary Table S1) in tumor development, differential analysis was performed evaluating SLCs gene expression in each TCGA tumor type (*N* = 33) compared to the pooled GTEx control group. The analysis identified 331 DEGs in at least 1 tumor type (log_2_FC ≥ 2 or log_2_FC ≤ −2; *p* ≤ 0.05) (Figure 1A; Supplementary Table S2). Interestingly, the *SLC2A2*, *SLC18A1*, *SLCO1B1*, *SLC24A5*, *SLC34A2*, *SLC4A1*, *SLC22A2*, *SLC17A3*, and *SLC22A7* were strongly upregulated (log_2_FC ≥ 10) in different tumor types including LIHC, PCPG, UVM, THCA, LUAD, KICH, and KIRC. Conversely, *SLC58A2*, *SLC26A10*, *SLCO2B1*, *SLC1A1*, and *SLC6A1* genes were strongly downregulated (log_2_FC ≤ −6) in LAML (Figure 1A; Supplementary Table S2).

To select the main SLCs dysregulated in cancer with respect to pooled GTEx control group, the number of tumors (*N* ≥ 15) in which DEGs showed log_2_FC higher than 2, 4, and 6 or less than −2, −4, and −6 was calculated (Figure 1B; Supplementary Table S2). According to these criteria, the SLC genes downregulated (log_2_FC ≤ −2, *p* ≤ 0.05) were 33 including *SLC25A27* (*N* = 29), *SLC17A7* (*N* = 27), *SLC26A10* (*N* = 26), *SLC2A4* (*N* = 26), *SLC42A1 (N = 26)*, and *SLC6A1*(*N* = 24) that showed the highest numbers of tumors (Figure 1B; Supplementary Table S2). Among these, *SLC26A10* and *SLC2A4* showed log_2_FC ≤ −4 in 11 and 8 tumor types, respectively. Furthermore, only *SLC26A10* and *SLC6A1* genes displayed log_2_FC ≤ −6 in LAML. Conversely, only 8 SLCs were upregulated (log_2_FC ≥ 2, *p* ≤ 0.05) in more than 14 tumors reaching 20 for *SLC27A2*. Among these, *SLC44A4*, *SLC34A2*, and *SLC6A14* showed log_2_FC ≥ 4 in at least 9 tumor types and log_2_FC ≥ 6 in more than 5 tumors (Figure 1B; Supplementary Table S2).

**FIGURE 2:**
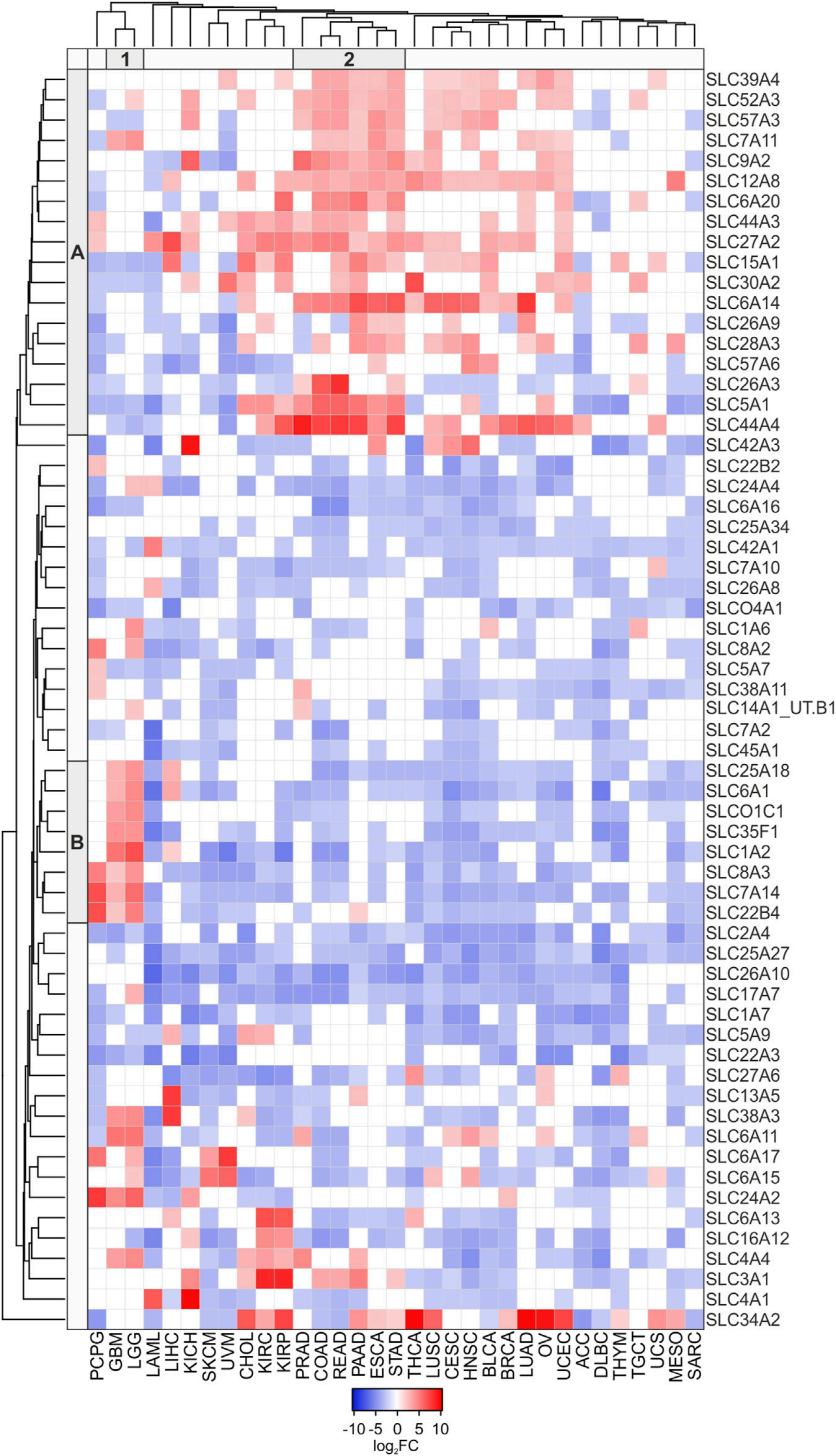
Heatmap of DEGs SLC (log2FC ≥ 2 or log2FC ≤ −2; *p* ≤ 0.05) in at least 15 tumor types compared to pooled GTEx control group. Average linkage clustering method and the Euclidian distance measurement method were applied according to Heatmapper software (http://heatmapper.ca). The red and blue squares indicate the up- and downregulated genes, respectively. The letters A and B (row) and the number 1 and 2 (column) identify the 2 main Clusters.

Among the 331 DEGs in TCGA tumors compared to the pooled GTEx control group, clustering analysis was performed on 62 SLCs showing either log_2_FC ≥ 2 or ≤ −2 in at least 15 tumor types (Figure 2; Supplementary Table S2). The results revealed an interesting cluster of SLC genes strongly upregulated (log_2_FC ≥ 2; *p* ≤ 0.05) in PRAD, COAD, READ, PAAD, ESCA, and STAD. In particular, the highest values of log_2_FC were observed for *SLC44A4* (9.53) in PRAD, *SLC26A3* (8.30) in READ, and *SLC6A14* (7.41) in PAAD (Figure 2, Cluster A2). Furthermore, the Cluster B1 grouped the *SLC25A18*, *SLC6A1*, *SLCO1C1*, *SLC35F1*, *SLC1A2*, *SLC8A3*, *SLC7A14*, and *SLC22B4* genes that were all upregulated (log_2_FC range: 2.12–7.13) in LGG and GBM (Figure 2; Supplementary Table S2).

**FIGURE 3:**
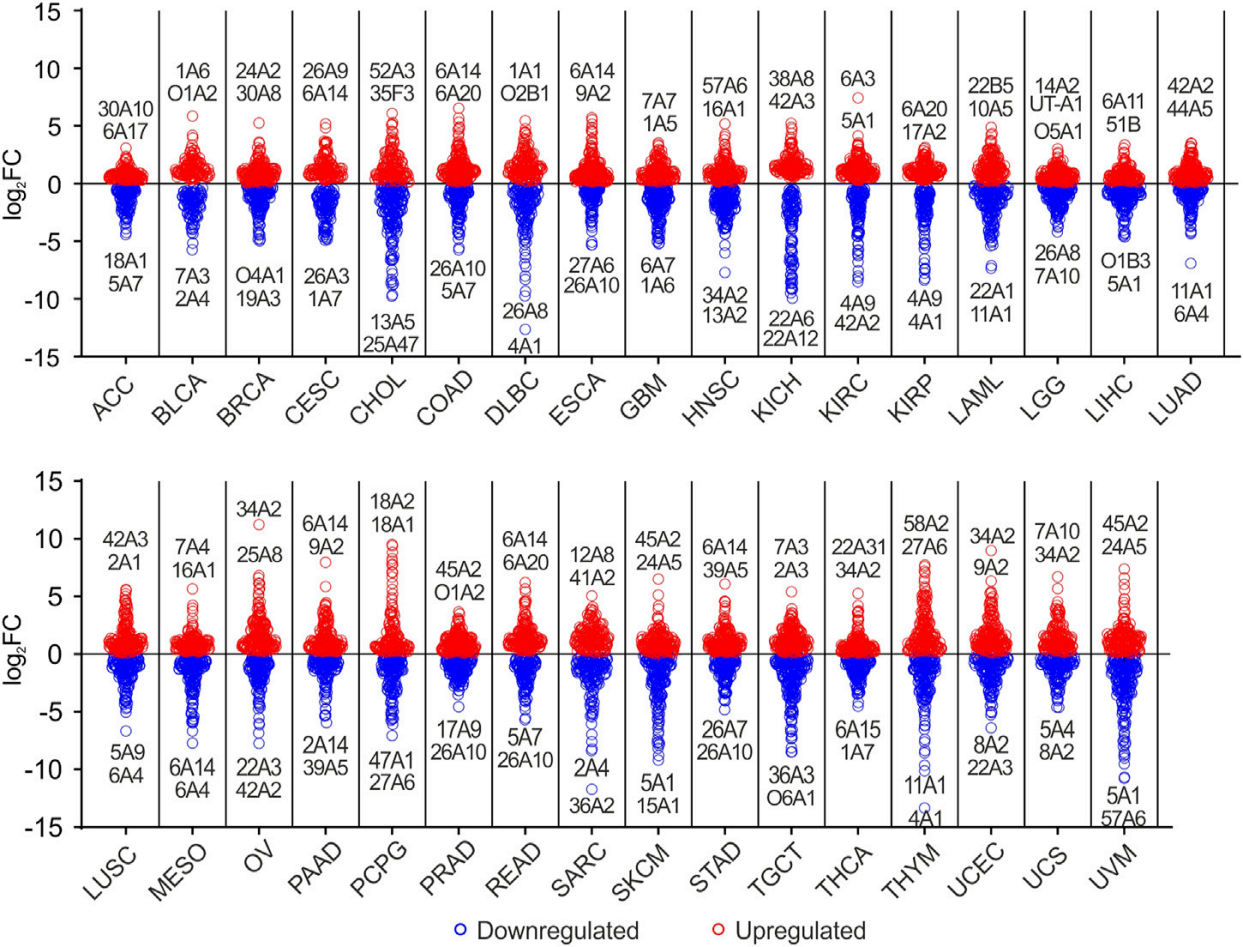
Graphical representation of DEGs SLC in TCGA tumors compared to matched normal tissue. Blue dots indicate the downregulated SLCs, whereas red dots represent the upregulated genes (*p* ≤ 0.05). Top two down- and upregulated SLC genes and relative tumor type were labeled. For each SLC gene is reported only the family, subfamily and isoform integers.

To better investigate DEGs SLC taking into account the tissue origin of each tumor type, differential analysis was conducted comparing each TCGA tumor with matched normal tissue (Table 1). The results indicated that 30 SLCs showed log_2_FC ≥ 6 in 13 tumor types (Figure 3; Supplementary Table S3). In particular, *SLC34A2* showed the highest log_2_FC (11.23) in OV, followed by *SLC18A2* and *SLC18A1* in PCPG, and *SLC34A2* in UCEC. Of note, *SLC6A14* was strongly upregulated (log_2_FC ≥ 6) in 4 gastrointestinal tumors, including COAD, PAAD, READ, and STAD. Conversely, 91 SLCs were significantly downregulated (log_2_FC ≤ - 6) in more than 50% of tumors (*N* = 18). Notably, *SLC4A1* (THYM and DLBC), *SLC36A2* (SARC), *SLC57A6*, *SLC15A1,* and *SLC5A1* (UVM), and *SLC11A1* (THYM) showed the lowest log_2_FC (≤−10) (Figure 3;Supplementary Table S3). Among the top two DEGs SLC, *SLC6A14* was upregulated in 6 tumor types, whereas *SLC26A10* was downregulated in 5 tumors (Figure 3; Supplementary Table S3). Both these SLCs were among the most represented DEGs (≥15 tumor types) obtained by differential analysis between each TCGA tumor and the pooled GTEx control group (Figure 1B, Figure 2).

### 3.2 OS and PFI analyses according to SLCs expression

**FIGURE 4:**
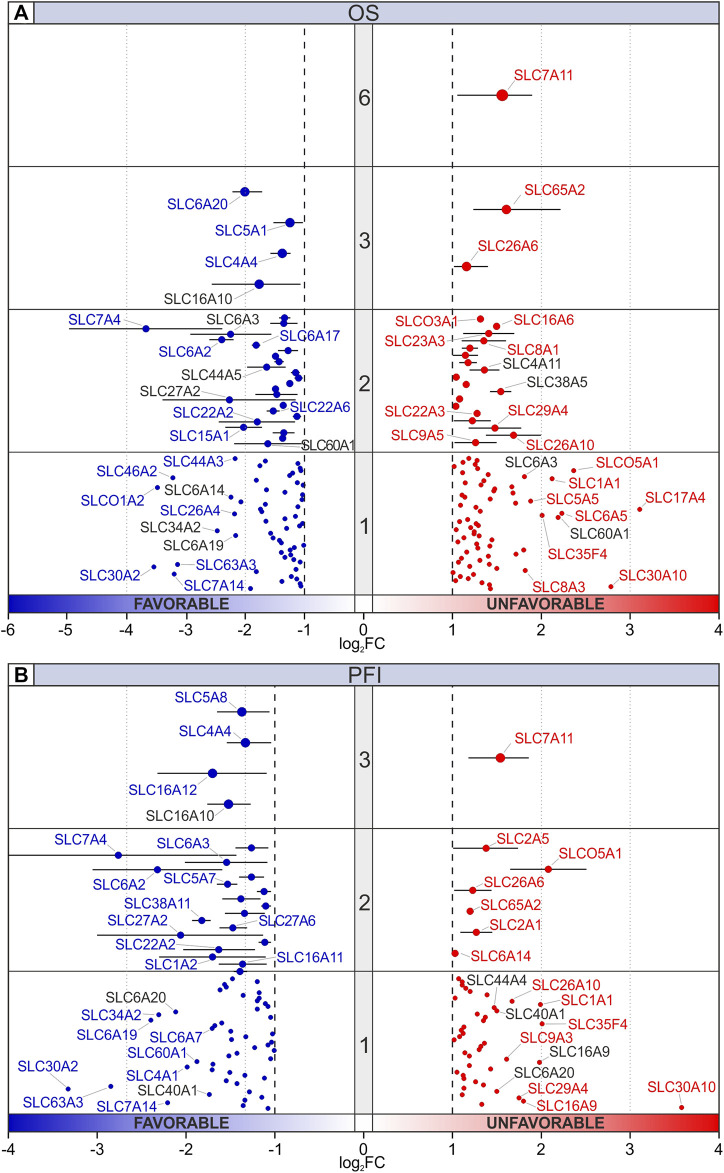
Prognostic significance of DEGs SLC. **(A)** OS and **(B)** PFI analyses of SLC genes stratified for tumor types comparing dead and alive TCGA Pan-cancer patients at 5 years. Median, maximum, and minimum value of log_2_FC for each DEG (log_2_FC ≥ 1 or ≤ −1, *p* ≤ 0.05) and number of tumor types (central grey band and dot size) were represented. Red or blue labeled SLCs indicate up- or downregulated genes, respectively. Black labels indicate SLCs that are not concordant.

OS analysis was performed to compare the expression levels of each SLC gene between dead and alive patients at 5 years and stratified for tumor type (Figure 4A; Supplementary Figure S1A; Supplementary Table S4). OS DEGs with log_2_FC ≥ 1 or ≤ −1 (*p* ≤ 0.05) were represented in Figure 4A including also the number of tumor types in which each gene was differentially expressed (central grey band and dot size). The analysis revealed that only *SLC7A11* was negatively associated with OS in 6 tumors (ACC, KICH, KIRP, PCPG, THYM, and UVM). Similarly, *SLC65A2* and *SLC26A6* were significantly associated with poor prognosis in 3 tumor types including KICH and PRAD for both SLC genes, KIRP for *SLC65A2*, and ACC for *SLC26A6*. The highest log_2_FC was observed for *SLC17A4* and *SLC30A10*, however both genes were negatively correlated with OS in one tumor (PRAD and ACC, respectively) (Figure 4A; Supplementary Table S4). On the other hand, *SLC6A20*, *SLC5A1*, *SLC4A4*, and *SLC16A10* were positively associated with OS in 3 tumor types. Among SLC genes with favorable significance in 2 tumors, *SLC7A4* showed the lowest log_2_FC in ACC (log_2_FC = −4.98) and PRAD (log_2_FC = −2.39). Interestingly, *SLC30A2* and *SLCO1A2* were strongly associated with favorable OS in ACC and THYM, respectively (Figure 4A; Supplementary Table S4). Supplementary Figure S1A shows the distribution of SLC genes positively (log_2_FC ≤ −1) or negatively (log_2_FC ≥ 1) associated with OS in all tumor types. The analysis revealed that the highest percentage of SCL genes negatively associated with OS was observed for UVM (16%), THYM (14%), ACC (13%), KIRP (12%), and MESO (11%). Conversely, the pro-survival SLC genes were mainly represented in ACC (17%), KIRC (13%), THYM (13%), and PCPG (11%) (Supplementary Figure S1A).

The impact of SLCs expression on PFI was analyzed at 5 years for each tumor type (Figure 4B; Supplementary Figure S1B; Supplementary Table S4). The analysis revealed that *SLC7A11*, *SLCO5A1*, and *SLC30A10* were the highest significant upregulated DEGs, which are predictive of a worse prognosis. In particular, *SLC7A11* was an unfavorable factor in ACC, KICH, and KIRP (Figure 4B; Supplementary Table S5). Conversely, *SLC6A2*, *SLC7A4*, and *SLC30A2* were the most significant DEGs associated with a long-lasting PFI. Among tumor types, ACC showed the highest number of SLCs influencing the progression of disease (Supplementary Figure S1B; Supplementary Table S5). Notably, OS and PFI were concomitantly affected by several SLCs including *SLC7A11*, *SLC65A2*, *SLC26A6*, and *SLC30A10* as unfavorable factors, and *SLC6A20*, *SLC7A4*, and S*LC30A2* as favorable ones (Figure 4).

### 3.3 Immune phenotype profile and differential analysis of SLCs gene expression in TCGA tumors

**FIGURE 5:**
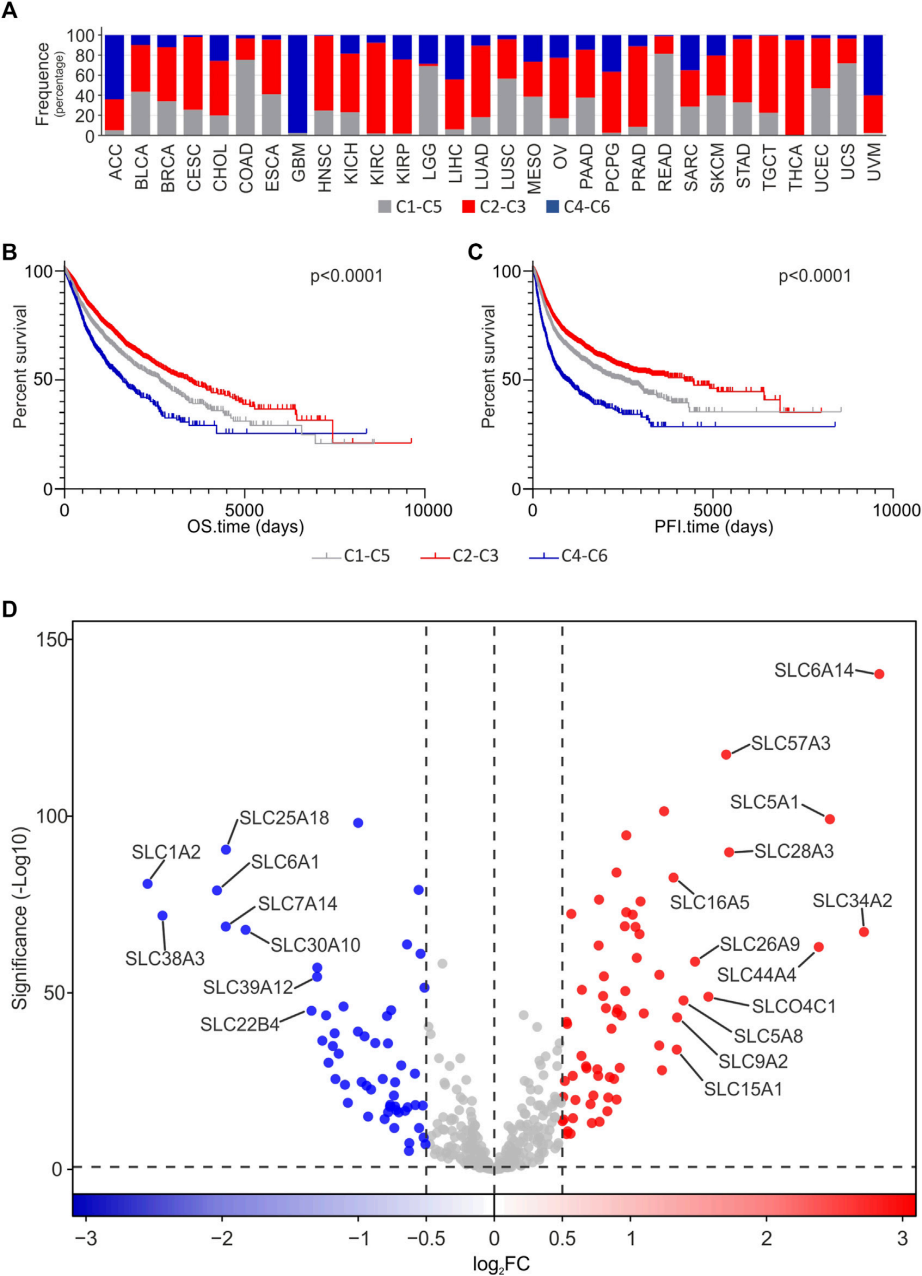
Differential analysis of SLCs gene expression according to cancer immune phenotype. **(A)** Frequency distribution of C1-C5, C2-C3, and C4-C6 immune phenotype groups among TGCA Pan-cancer tumor types. OS **(B)** and PFI **(C)** Kaplan Meier analyses performed stratifying cancer patients according to C1-C5, C2-C3, and C4-C6 immune phenotype groups. **(D)** Volcano plot of SLC DEGs in C2-C3 immune signature compared to C4-C6 immune signature in all TCGA Pan-cancer tumor types. Red and Blue dots represent up- and down-regulated genes, respectively (log_2_FC ≥ 0.5 or ≤ −0.5, *p* ≤ 0.05). The top 20 down- and upregulated SLC genes showing both the highest significance and log_2_FC are labeled. The *p*-value is represented as -Log10.

To evaluate the potential association between SLCs gene expression and the intratumoral immune state of each tumor sample, differential analysis was performed stratifying tumor samples in 3 groups based on their immune signatures (Figure 5). In particular, the immuno-quiet (C4-C6) group included the C4 and C6 tumoral signatures, the immune-response group (C2-C3) included the C2 and C3 signatures, while the third group (C1-C5) comprised the C1 and C5 signatures. These signatures were retrieved from “Subtype_Immune_Model_Based” in analytic data type available in Xena UCSC provided by Thorsson et al. (2018). The distribution analysis of immune groups revealed that the C2-C3 group was the most represented in most of tumor types, except for ACC, GBM, and UVM in which the C4-C6 group showed the highest percentage. As regard C1-C5 immune group, it was mainly represented in COAD, LGG, LUSC, READ, and UCS (Figure 5A). Interestingly, OS and PFI analyses indicated that cancer patients belonged to immune quiet group (C4-C6) had a lower percent survival compared to immune response group (C2-C3), which was strongly associated to a better prognosis. The C1-C5 group showed intermediate OS and PFI rates compared the other two immune phenotypes (Figures 5B, C. To identify the DEGs SLC involved in immune response, differential analysis of SLC genes was performed between immune quiet (C4-C6) and immune response (C2-C3) groups, as the main intratumoral immune phenotypes affecting the clinical outcome. The results indicated that 65 SLCs were upregulated in the C2-C3 group, whereas 56 were downregulated (Figure 5D; Supplementary Table S6). Interestingly, *SLC6A14*, *SLC34A2*, *SLC5A1*, and *SLC44A4* showed log_2_FC ≥ 2 in tumors with C2-C3 signature. Furthermore, these SLC genes were strongly upregulated (log_2_FC ≥ 2) in tumors compared to pooled GTEx control group, except for *SLC5A1* (Figure 1B; Supplementary Table S2). Interestingly, *SLC6A14*, *SLC34A2*, and *SLC5A1* also resulted among the top DEGs SLC when comparing each TCGA tumor type with matched normal tissue (Figure 3; Supplementary Table S3). Conversely, *SLC6A1*, *SLC38A3*, and *SLC1A2* were negatively associated (log_2_FC ≤ −2) to C2-C3 signature (Figure 5D; Supplementary Table S6). Interestingly, all of these three genes were strongly downregulated in almost half of the TCGA tumors compared to pooled GTEx control group (Figure 1B; Supplementary Table S2).

### 3.4 Correlation of SLCs expression and drug sensitivity in cancer cell lines

CCLE dataset was explored to identify the SLC genes involved in drug response of several cancer cells (*N* = 504). The IC_50_ of 24 anticancer drugs was retrieved along with the expression levels (RPKM) of SLC genes (Supplementary Table S7).

**FIGURE 6:**
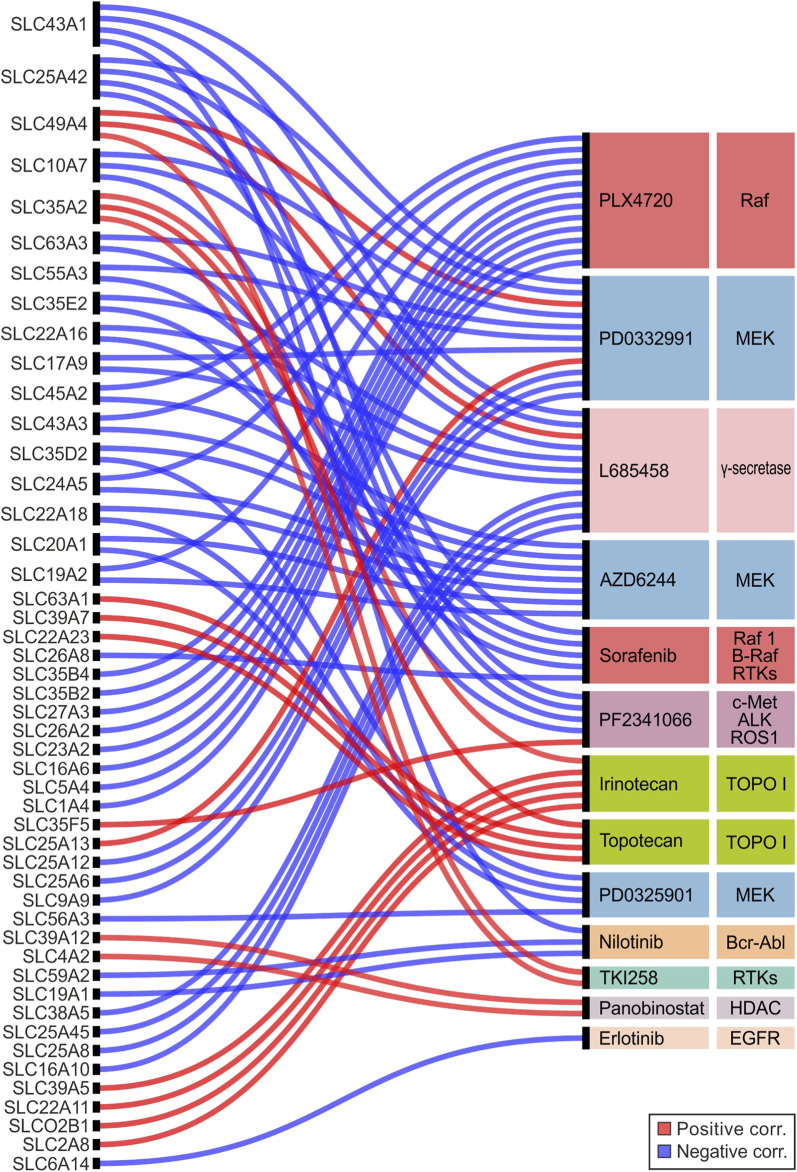
Alluvial plot analysis of the SLC genes significantly correlated (r ≤ −0.25 or r ≥ 0.25; *p* ≤ 0.05) with IC_50_ of anticancer drugs tested in 504 CCLE cell lines. Red and blue lines indicated positive and negative correlations, respectively. SLC genes and drugs were listed from top to bottom according to the number of interactions. The main targets of each drug were also indicated.

The results showed that 48 SLC genes were significantly correlated (r ≤ −0.25 or r ≥ 0.25, *p* ≤ 0.05) with the IC_50_ of 13 drugs. Among these PLX4720, PD0332991, and L685458 compounds correlated with more than 10 SLC genes (Figure 6; Supplementary Table S7). Notably, PLX4720, AZD6244, Sorafenib, PD0325901, and Nilotinib showed only negative correlations with SLCs gene expression, whereas Irinotecan, Topotecan, TKI258, and Panobinostat showed exclusively positive correlations. Regarding the SLC genes, the highest amount of correlations (*N* > 2) was observed for *SLC43A1*, *SLC25A42*, *SLC49A4*, *SLC10A7*, and *SLC35A2*. Of these only *SLC49A4* and *SLC35A2* were always positively correlated, while the other ones were negatively correlated (Figure 6; Supplementary Table S7). Among the SLCs significantly correlated with MEK and Raf inhibitors, the *SLC35D2*, *SLC20A1*, and *SLC22A18* showed negative correlation with both AZD6244 and PD0325901 (MEK inhibitors), whereas *SLC24A5*, *SLC45A2*, *SLC43A3*, and *SLC19A2* negatively correlated with both AZD6244 (MEK inhibitor) and PLX4720 (Raf inhibitor) (Figure 6; Supplementary Table S7). Remarkably, both *SLC24A5* and *SLC45A2* were strongly upregulated (log_2_FC > 8) in SKCM and UVM when compared to both matched normal tissue and pooled GTEx control group, for which the elective pharmacological strategy is based on MAPK inhibitors treatment (Figure 1A, Figure 3; Supplementary Tables S2, S3). The Erlotinib showed only one negative correlation with the *SLC6A14* gene (Figure 6; Supplementary Table S7) that was upregulated in 15 tumor types including PRAD compared to pooled GTEx control group (Figure 1B; Supplementary Table S2), as well as in tumors with C2-C3 signature (Figure 5D; Supplementary Table S6). Moreover, *SLC6A14* was among the top two upregulated SLC genes in 6 tumor types (PAAD, COAD, READ, STAD, ESCA, and CESC), while it was downregulated in MESO when performed differential analysis between each tumor type and matched normal tissue (Figure 3; Supplementary Table S3). Interestingly, this SLC was also downregulated (log_2_FC ≤ −2) in 5-year dead PRAD patients (Figure 4A; Supplementary Table S4). Collectively, these results suggested that *SLC6A14* overexpression is associated to better outcome for prostate cancer patients.

### 3.5 DNA methylation profiling of relevant SLC genes in TCGA pan-cancer

**FIGURE 7:**
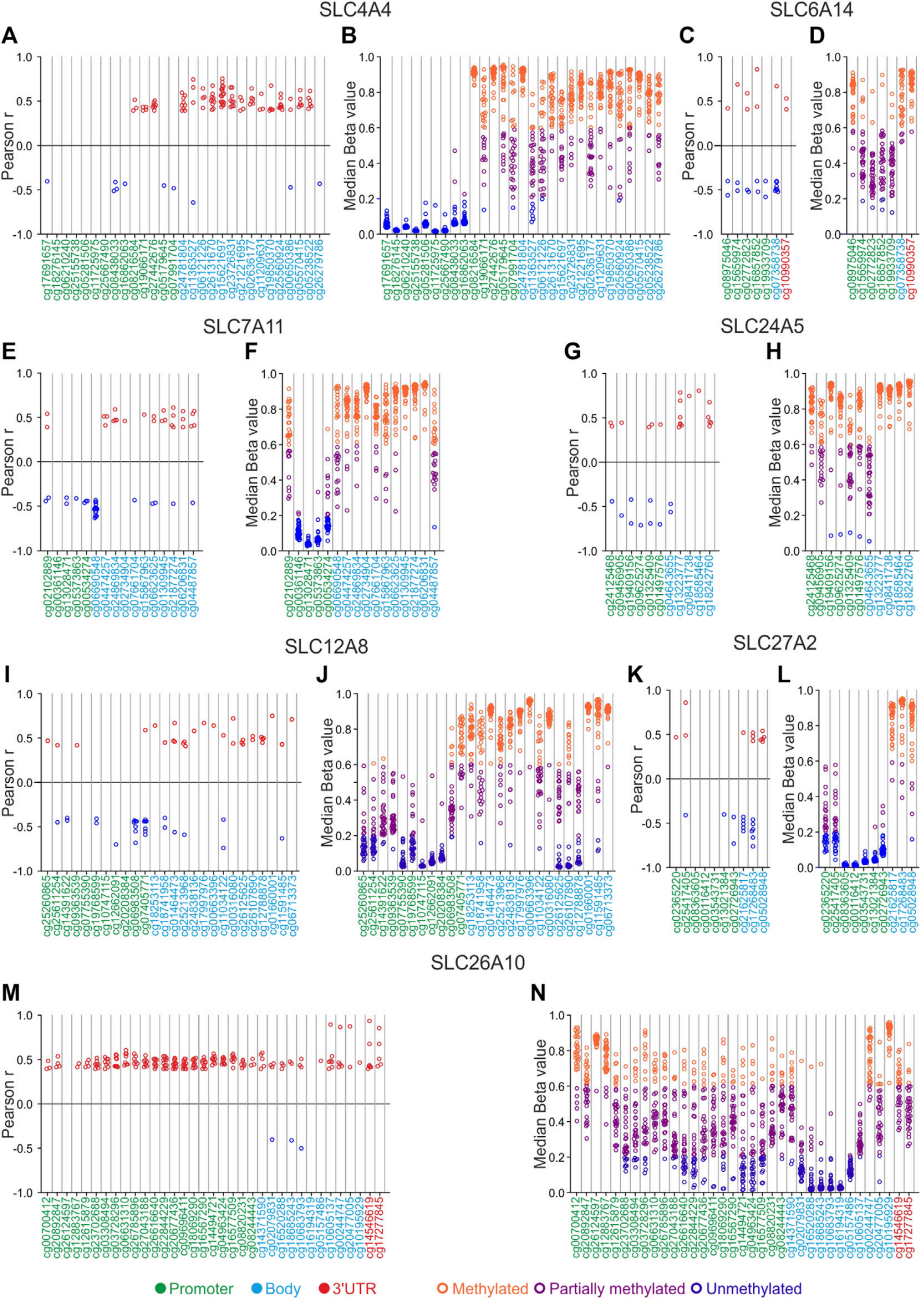
SLCs correlation and DNA methylation status analyses. **(A,C,E,G,I,K,M)** Correlation pairs showing Pearson’s r ≥ 0.4 or ≤ −0.4 are indicated as red or blue dots, respectively. **(B,D,F,H,J,L,N)** Median Beta value of CG probesets was calculated for each TCGA Pan-cancer tumor type. Orange dots indicate the tumor types in which each CG probeset is methylated, purple dots indicate those showing partially methylated CG probesets, while blue dots indicate hypomethylation. The position of each CG probeset within the promoter, body, and 3′UTR regions was indicated by green, cyan, and red labeling, respectively.

**FIGURE 8:**
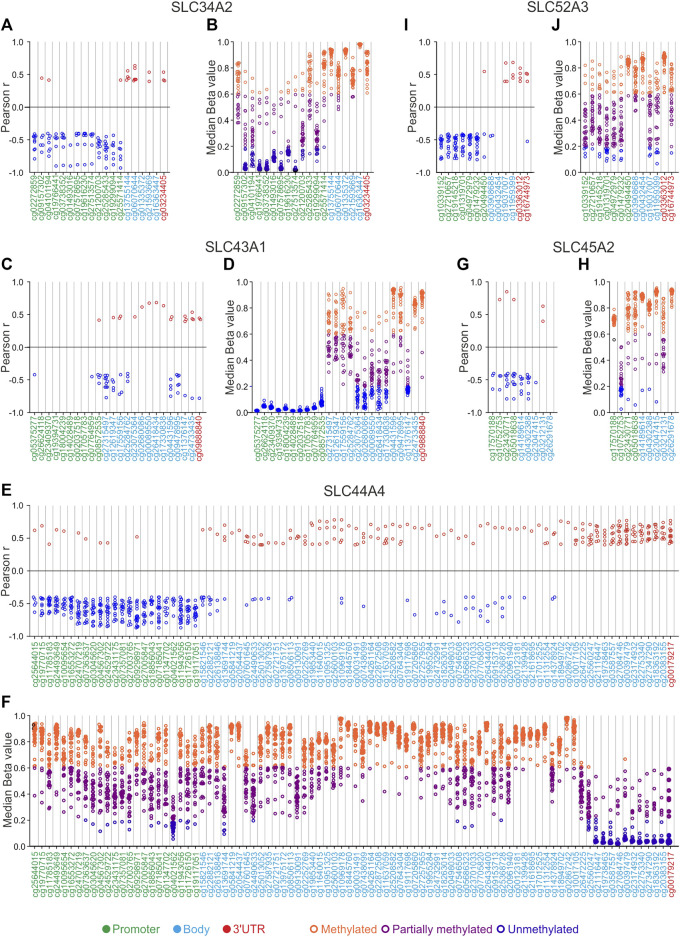
SLCs correlation and DNA methylation status analyses. **(A,C,E,G,I)** correlation pairs showing Pearson’s r ≥ 0.4 or ≤ −0.4 are indicated as red or blue dots, respectively. **(B,D,F,H,J)** median Beta value of CG probesets was calculated for each TCGA Pan-cancer tumor type. Orange dots indicate the tumor types in which each CG probeset is methylated, purple dots indicate those showing partially methylated CG probesets, while blue dots indicate hypomethylation. The position of each CG probeset within the promoter, body, and 3′UTR region was indicated by green, cyan, and red labeling, respectively.

To evaluate the role of DNA methylation in gene expression of main SLC genes involved in tumorigenesis, survival, immunity and drug resistance (Table 2), the correlation analysis between gene expression and DNA methylation levels of these genes was performed in each of the 33 TCGA Pan-Cancer tumors (Figures 7, 8; Supplementary Table S8). Of note, correlation and DNA methylation analyses were conducted on the top 12 DEGs SLC (6 up- and 6 downregulated retrieved from differential analysis between each TCGA tumor type and pooled GTEx control group), 4 SLCs strictly related to OS and PFI, 4 SLCs implicated in tumoral immune response, and 6 SLCs mainly associated to drug sensitivity (Table 2). However, the correlation plots were only reported for the SCLs showing ≥50% of correlated CG probesets for at least one tumor type or at least one CG significantly correlated (r ≥ 0.4 or r ≤ −0.4; *p* ≤ 0.05) in more than half of Pan-Cancer tumor types (Figures 7, 8; Table 2). Furthermore, the DNA methylation status of the selected SLCs was evaluated by calculating the median values of relative CG probesets for each tumor type (Figures 7, 8; Supplementary Figure S2; Supplementary Table S9).

**TABLE 2:** Main SLC genes involved in tumorigenesis, survival, immunity, and drug resistance analyzed for correlation between gene expression and DNA methylation. The maximum number of CG probesets showing correlation with gene expression was calculated for each gene in all TCGA Pan-Cancer tumors. Furthermore, the number of tumors showing significant correlation pairs (r ≥ 0.4 or r ≤ −0.4, *p* ≤ 0.05) was evaluated for CG probesets of each relevant SLC.

SLC gene	Main tumoral characteristics related to SLCs	Number of correlation pairs	
		N. Max of CGs per tumor	N. Max of tumors per CG
*SLC1A2*	**Immune Response-,** Tumorigenesis↑, PFI+	6 of 28 (21.4%)	6 of 33
*SLC2A4*	**Tumorigenesis↓**	3 of 16 (18.8%)	4 of 33
*SLC4A4* ^a^	**OS+ and PFI+**	15 of 29 (51.7%)	18 of 33
*SLC6A1*	**Tumorigenesis↓** and Immune Response-	12 of 33 (36.4%)	3 of 33
*SLC6A14* ^a^	**Tumorigenesis↑**, **Immune Response+**, DRUG sensitivity+, PFI- and OS+	6 of 7 (85.7%)	10 of 33
*SLC7A11* ^a^	**OS- and PFI-**	6 of 16 (37.5%)	17 of 33
*SLC10A7*	**DRUG sensitivity+**	7 of 24 (29.2%)	3 of 33
*SLC12A8* ^a^	**Tumorigenesis↑**	16 of 26 (61.5%)	8 of 33
*SLC16A10*	**OS+, PFI+**, and DRUG sensitivity+	1 of 13 (7.7%)	2 of 33
*SLC17A7*	**Tumorigenesis↓**	13 of 33 (39.4%)	5 of 33
*SLC24A5* ^a^	**DRUG sensitivity+**	7 of 11 (63.6%)	7 of 33
*SLC25A27*	**Tumorigenesis↓**	6 of 15 (40.0%)	8 of 33
*SLC25A42*	**DRUG sensitivity+**	4 of 20 (20.0%)	9 of 33
*SLC26A10* ^a^	**Tumorigenesis↓**, OS-, and PFI-	23 of 35 (65.7%)	12 of 33
*SLC27A2* ^a^	**Tumorigenesis↑**, OS+ and PFI+	5 of 10 (50.0%)	8 of 33
*SLC34A2* ^a^	**Tumorigenesis↑** and **Immune Response+**, OS+ and PFI+	14 of 19 (73.7%)	11 of 33
*SLC35A2*	**DRUG sensitivity-**	3 of 18 (16.7%)	2 of 33
*SLC38A3*	**Immune Response-** and Tumorigenesis↓	4 of 15 (26.7%)	8 of 33
*SLC42A1*	**Tumorigenesis↓**	1 of 4 (25%)	1 of 33
*SLC43A1* ^a^	**DRUG sensitivity+**	13 of 24 (54.2%)	10 of 33
*SLC44A4* ^a^	**Tumorigenesis↑** and Immune Response+	65 of 88 (79.9%)	20 of 33
*SLC45A2* ^a^	**DRUG sensitivity+**	7 of 9 (77.8%)	9 of 33
*SLC49A4*	**DRUG sensitivity-**	7 of 20 (35%)	3 of 33
*SLC52A3* ^a^	**Tumorigenesis+**	12 of 13 (92.3%)	17 of 33
*SLC65A2*	**OS- and PFI-**	6 of 16 (37.5%)	1 of 33

In bold are represented the tumor characteristics used to select relevant SCL genes.

^a^ Pearson’s correlation graph available.

**↑**: upregulated; ↓: downregulated; **+**: positively associated; -: negatively associated.

The correlation analysis between DNA methylation levels of CG probesets and gene expression of the *SLC4A4* gene revealed that correlation pairs were mainly observed in the body CG probesets showing a positive correlation in several tumor types (*N* = 18 for cg15621697) (Figure 7A; Supplementary Table S8). As regards the *SLC6A14*, 6 of 7 CG probesets were negatively correlated with gene expression in PRAD (Figure 7C; Supplementary Table S8). Interestingly, the cg06690548 probeset of *SLC7A11* body region, immediately nearby the promoter region, showed the highest number of negative correlation pairs (*N* = 17) (Figure 7E; Supplementary Table S8). The correlation analysis of *SLC24A5* revealed that the methylation status of all promoter CG probesets as well as the cg04643655 body probeset, was negatively correlated (r from −0.44 to −0.71; *p* ≤ 0.05) with the *SLC24A5* expression only in SKCM (Figure 7G; Supplementary Table S8). Moreover, all the CG probesets within the body region of *SLC12A8* were positively correlated with gene expression in several tumor types (Figure 7I; Supplementary Table S8). Similarly, *SLC27A2* was positively correlated with 2 of 7 promoter CG probesets and all body CG probesets only in TGCT (Figure 7K; Supplementary Table S8). Surprisingly, the correlation analysis of *SLC26A10* displayed a positive correlation between gene expression and DNA methylation levels for all CG probesets in a large number of tumors. In particular, the promoter region (from cg23702688 to cg16577509) showed the highest number of correlation pairs in ≥7 tumor types. Notably, ACC, BLCA, ESCA, SARC, and UCEC showed the highest number of positive correlated CG probesets (*N* ≥ 18) (Figure 7M; Supplementary Table S8).

DNA methylation analysis revealed that *SLC4A4* CG probesets of the distal promoter region were hypomethylated (median beta value ≤0.2) in all Pan-Cancer tumor types (Figure 7B; Supplementary Table S9). A similar trend was observed for some promoter CG probesets of both *SLC7A11* (from cg00361146 to cg00534274), *SLC12A8* (cg10774115, cg12662091, and cg20208384), and *SLC27A2* (from cg02365220 to cg02726943) (Figures 7 F, J, L; Supplementary Table S9). In addition, the other CG probesets mainly located in the body and 3′UTR regions of *SLC4A4*, *SLC6A14*, *SLC7A11*, *SLC24A5*, *SLC12A8*, and *SLC27A2*, as well as the first CG probeset of *SLC6A14* and *SLC7A11* promoter region, were partially or markedly methylated (Figures 7B, D, F, H, J, L). Interestingly, the *SLC24A5* CG probesets from cg19409156 to cg04643655 were unmethylated in UVM (Figure 7H; Supplementary Table S9).

Regarding the methylation status of *SLC26A10*, the median levels of CG probesets showed a broad distribution of beta values (from 0.01 to 0.96) among the analyzed tumor types, except for some CG probesets of body region (from cg02079831 to cg05157486) that were strongly hypomethylated (Figure 7N; Supplementary Table S9). Similarly, all the promoter CG probesets of *SLC24A5* were partially or hyper-methylated (Figure 7H; Supplementary Table S9).

For the last group of the selected SLCs listed in Table 2, the correlation analysis revealed that both *SLC34A2* (Figure 8A; Supplementary Table S8) and *SLC44A4* (Figure 8E; Supplementary Table S8) showed a cluster of negative correlated CG probesets in the promoter region. Notably, OV, KIRP, and THCA were the most representative tumors with the highest negative correlation values for *SLC34A2* (r ≤ −0.70; *p* ≤ 0.05), whereas BRCA, ESCA, KIRP, and CHOL showed ≥10 promoter CG probesets negatively correlated (r ≤ −0.70; *p* ≤ 0.05) with the *SLC44A4* expression (Figures 8 A, E; Supplementary Table S8). Concomitantly, the last CG probesets of *SLC44A4* body region (from cg25560247 to cg20383155) and the 3′UTR CG probeset showed a cluster of positive correlated pairs (r ≥ 0.4; *p* ≤ 0.05), especially for CESC, COAD, DLBC, ESCA, KIRP, PAAD, and READ (≥10 GC probesets *per* tumor type) (Figure 8E; Supplementary Table S8). Correlation analysis of *SLC45A2* revealed a predominant negative correlation in most of CG probesets (Figure 8G; Supplementary Table S8). Similarly, *SLC43A1* showed a higher number of negative correlation pairs compared to positive ones localized both in the body and 3′UTR regions, while no significant correlation was observed within the promoter region (Figure 8C; Supplementary Table S8). Conversely, the promoter region of the *SLC52A3* showed a negative correlation with its expression levels in at least 12 tumor types, except for cg20494450 (Figure 8I; Supplementary Table S8). The methylation profiles of SLC34*A2*, *SLC45A2*, and *SLC52A3*, as well as most of CG probesets of *SLC43A1* and *SLC44A4*, showed that the DNA methylation levels gradually varied over a wide range in TCGA Pan-cancer tumors (Figures 8B, D, F, H, J; Supplementary Table S9). Interestingly, the CG probesets of *SLC43A1* were collectively unmethylated in the promoter region for all tumor types (Figure 8D; Supplementary Table S9). A similar trend was observed for the last *SLC44A4* CG probesets of the body region (from cg25560247 to cg20383155) and the 3′UTR CG probeset that were mainly unmethylated or partially methylated (Figure 8F; Supplementary Table S9).

The SLCs strictly related to DNA methylation, their biological functions and involvement in main cancer features are summarized in Supplementary Figure S3.

### 3.6 Prognostic significance of cg06690548 methylation levels

**FIGURE 9:**
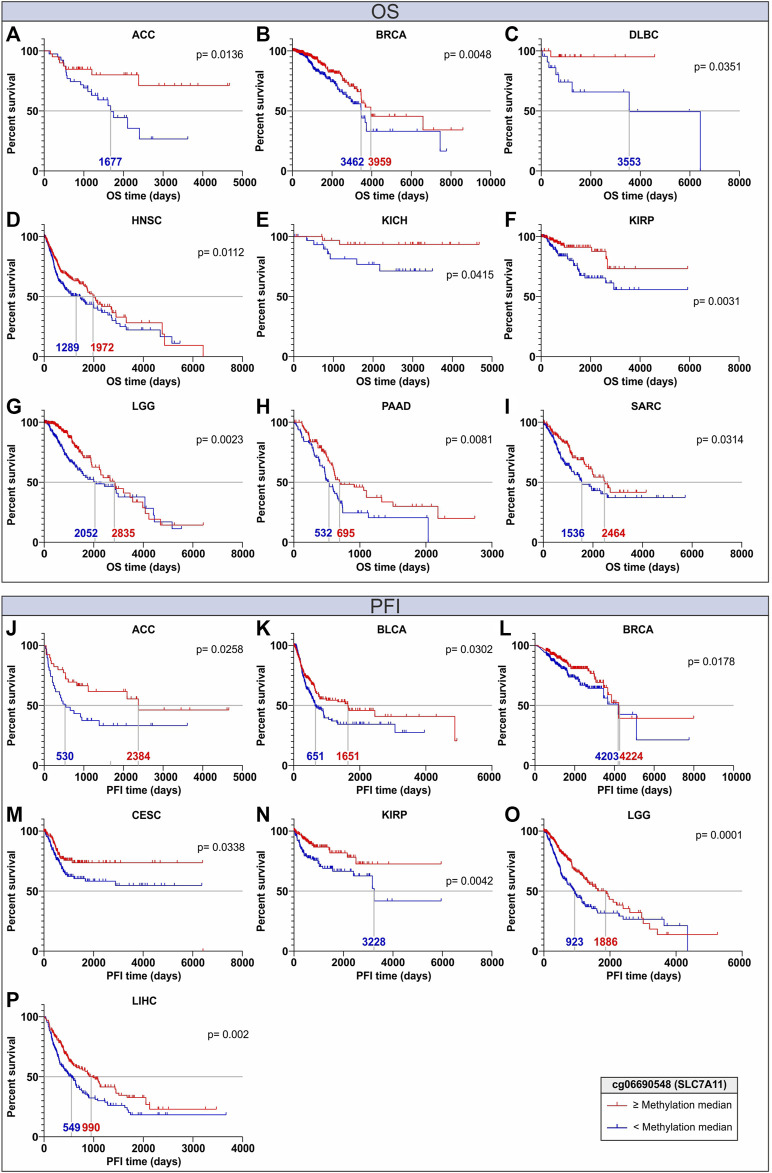
Kaplan Maier analyses for OS **(A–I)** and PFI **(J–P)** of cancer patients stratified according to DNA methylation levels of the cg06690548 probeset within the early body region of *SLC7A11*. The curves for the high and low DNA methylation groups are indicated in red and blue, respectively. The analyses were performed for each TCGA Pan-cancer tumor type; however, only significant (*p* ≤ 0.05) Kaplan Maier plots are shown. The median of OS and PFI time is also reported for each curve.

To evaluate if the DNA methylation of SLC genes associated with OS and PFI can be proposed as an independent prognostic biomarker, Kaplan Meier (KM) analysis was performed in all TCGA Pan-cancer tumor types stratifying the patients according to the cg06690548 methylation levels of the *SLC7A11* gene, which was found to be strictly associated with OS and PFI in several tumor types (Figure 4; Supplementary Tables S4, S5). Of note the cg06690548 probeset methylation was negatively correlated (r ≤ −0.4; *p* ≤ 0.05) with *SLC7A11* expression in 17 tumor types (Figure 7E; Supplementary Table S8). The KM analysis revealed that the patients with cg06690548 DNA methylation levels above the median values computed for all patients showed a better OS in 9 tumors, especially for ACC and DLBC that showed the highest distance between the survival curves (Figures 9A–I). In addition, the cg06690548 hypermethylation was positively associated with PFI in 7 tumor types with the best performance for ACC, KIRP, and LGG (Figures 9J–P).

## 4 Discussion

Overgrowth and uncontrolled proliferation are the most distinctive hallmarks of the cancer during carcinogenesis. The reprogramming of the biomolecules trafficking across the cellular membrane is a consequence of the increment of metabolic and biosynthetic pathways (Sneeggen et al., 2020). To overcome these new cell demands, an extensive remodulation of SLC genes occurs through different gene regulation mechanisms including DNA methylation (Bhutia et al., 2016). This epigenetic mark is a preferential mechanism involved in the long-term expression or silencing of key genes in cancer cells (Reddington et al., 2014). However, the role of many SLC genes in cancer has not yet been investigated and an overview on their dysregulation is mandatory to better clarify their involvement in tumor development. To this purpose, *in silico* study was performed to identify the role of all annotated SLC genes in tumorigenesis, survival, tumor immune profile, and drug response in main cancer types. The analyses were conducted using the gene expression data from TCGA Pan-cancer tumor and GTEx normal samples, whereas DNA methylation data were obtained from the TCGA Pan-cancer cohort. In particular, differential analysis was executed to assess the association of each SLC gene with the different clinic-pathological features of cancer types analyzed in this study. The filtering procedure allowed to identify the most relevant SLC genes (*N* = 25) strictly related to cancer for which DNA methylation analysis was performed. Specifically, the median levels of DNA methylation were evaluated for each CG probeset relative to the selected SLCs in each TCGA Pan-cancer tumor type. In addition, correlation analysis between DNA methylation levels of these CG probesets and gene expression of related SLCs was performed to identify the SLCs strictly modulated by DNA methylation across different cancer types (Table 2).

The obtained results allowed us to select 12 SLC genes that met the aforementioned criteria. Among these the *SLC4A4* gene was widely dysregulated in several tumor types when compared to pooled GTEx control group. These results are partially supported by the literature reporting the *SLC4A4* downregulation in COAD and upregulation in PAAD and PRAD (Chen et al., 2020; Liu Z. et al., 2022; Cappellesso et al., 2022). The *SLC4A4* gene encodes for a sodium-bicarbonate cotransporter involved in the regulation of bicarbonate secretion/absorption and intra/extra-cellular pH (Huynh et al., 2018). Recently, it has been demonstrated that the *SLC4A4* inhibition mitigated the acidosis of the TME in PAAD due to bicarbonate accumulation in the extracellular space and the reduction of the lactate secretion by cancer cells, which affected the T cell-mediated immune response and macrophage-mediated immunosuppression (Cappellesso et al., 2022). Moreover, our OS and PFI analyses showed that *SLC4A4* overexpression was a favorable prognostic factor in ACC, MESO, DLBC, and PCPG. Since the prognostic value of *SLC4A4* was mainly evaluated in COAD (Chen et al., 2020; Gao and Yang, 2020; Yang et al., 2020; Yang W. et al., 2022), this *in silico* evaluation could represent the starting point for the validation of its significance in other tumors.

The *SLC6A14* gene, a sodium/chloride-dependent neutral and cationic AAs transporter, was strongly overexpressed in several tumor types, including colorectal, gastric, pancreatic, breast, and cervical cancers (Mao et al., 2021; Guo et al., 2022; Schniers et al., 2022; Babu et al., 2015; Gupta et al., 2006). *In silico* analysis confirmed the *SLC6A14* overexpression in these tumors when compared to both matched normal tissue and pooled GTEx control group, as well as in other epithelial cancers, suggesting a positive correlation between its expression levels and cancer development. Mechanistically, the *SLC6A14* gene mediates the AAs uptake essential for the macromolecular synthesis and energy metabolism and modules the metabolic the mammalian target of rapamycin (mTOR) signaling (Guo et al., 2022; Lu et al., 2022). In addition, it was observed that *SLC6A14* is a preferential transporter of the same AA-base prodrugs (Bhutia et al., 2014). Since its selective overexpression in several tumors and its crucial role in cancer bioenergetic, *SLC6A14* could represent a suitable therapeutic target, as well as a specific drug carrier (Bhutia et al., 2014; Kou et al., 2020). Of note, the correlation analysis between *SLC6A14* and drug sensitivity in cancer cell lines revealed that SLC increases response to Erlotinib. However, these findings partially disagree with the literature that reports the role of *SLC6A14* in sustaining serine/glutathione-dependent drug resistance in cancer (Yoo and Han, 2022). Furthermore, we noted that *SLC6A14* was upregulated in intratumoral immune-response, supporting the evidence that immune cell activation is associated with metabolic reprogramming and an increase of molecular trafficking mediated by SLCs (Wang and Zou, 2020).

The bioinformatic analysis revealed that the expression of *SLC7A11* was a predictive factor of both worse OS and PFI in ACC, KICH, and KIRP. Of note, several studies have reported the negative prognostic significance in several tumor types, including pancreatic and lung cancers, mainly due to the antioxidant defense of cancer cells (Ji et al., 2018; Sharbeen et al., 2021). Indeed, the *SLC7A11* transporter mediates the cystine/glutamate antiporter leading to cystine uptake for the biosynthesis of glutathione, which represents a powerful reactive oxygen species (ROS) scavenger (Lee and Roh, 2022). Therefore, *SLC7A11* indirectly counteracts the polyunsaturated fatty acids oxidation enhancing the resistance to ferroptosis induced by anti-cancer drugs (Lin et al., 2020; Jyotsana et al., 2022).

Among the 12 SLCs, we found that the *SLC12A8* represents one of the most upregulated genes in cancer (TCGA tumor types vs*.* pooled GTEx control group differential analysis). This gene is a sodium-dependent nicotinamide mononucleotide (NMN) symporter that ensures the Nicotinamide Adenine Dinucleotide (NAD^+^) cell reservoir essential for energy metabolism (Grozio et al., 2019). Despite its crucial role in cell metabolism, the *SLC12A8* has been only investigated in bladder and breast (Li et al., 2020; Zhang Q. et al., 2021; Li et al., 2021).

Similarly, *SLC27A2* is the most upregulated gene comparing TCGA tumor types to pooled GTEx control group, especially in KIRC, COAD, KIRP, and LIHC. This gene encodes for a multifunctional protein, mainly localized in peroxisomes and endoplasmic reticulum, which activates and transports long-chain fatty acids involved in cancer lipid metabolism (Falcon et al., 2010; Qiu et al., 2020). However, opposite results are reported by Xu and colleagues, which highlighted that *SLC27A2* was downregulated in both renal cancer cell lines and tissues suggesting its correlation with favorable OS (Xu et al., 2022). This prognostic significance agrees with our OS and PFI analyses stratifying the KIRC patients according to *SLC27A2* expression levels. Therefore, further investigations are mandatory to better clarify the heterogeneous findings on *SLC27A2* as an emerging potential cancer biomarker.

According to our selection criteria, the anion transmembrane transporter *SLC26A10* emerged as a potential tumor suppressor factor due to its downregulation in more than 60% of the analyzed tumor types when compared to both matched normal tissue and pooled GTEx control group. However, to the best of our knowledge, few literature data are available on the *SLC26A10* physiological function and its relationship with cancer development.

*SLC34A2* gene encodes for pH-sensitive sodium-dependent phosphate transporter responsible for the transcellular inorganic phosphate absorption and surfactants synthesis in lung alveoli. It is normally expressed in various tissues, including lung, small intestine, and kidney (Wagner et al., 2014). Our computational analysis showed that the *SLC34A2* gene was upregulated in 15 tumor types when compared to pooled GTEx control group, especially in OV, THCA, and LUAD, whereas it was downregulated in 10, including COAD and BLCA. As reported in the literature, several studies have focused on OV, THCA, and LUAD, highlighting the positive correlation between *SLC34A2* and tumor development (Shyian et al., 2011; Wang et al., 2015; He et al., 2020). However, discordant results were observed for some tumors comparing our *in silico* data and literature. For instance, we found that *SLC34A2* was downregulated in COAD and BLCA, while previous studies reported that this transporter was upregulated in tumor tissues of colorectal and bladder cancer patients (Ye et al., 2017; Yang Y. et al., 2022). Interestingly, we found that *SLC34A2* was upregulated in immune-response (C2-C3) compared to immuno-quiet (C4-C6) signatures in tumor samples, suggesting its involvement into immunity activation. However, further studies should be performed to better clarify the underlying mechanism.

*SLC43A1*, also known as LAT3, belongs to a SLC family involved in the transport of neutral AAs, (leucine, isoleucine, valine, phenylalanine, and methionine) suggesting its involvement in mTOR leucine-dependent activation (Rii et al., 2021; Wang et al., 2021). Of note, our drug sensitivity analysis showed that *SLC43A1* overexpression sensitizes cancer cell lines to Nilotinib, Palbociclib (PD0332991), Crizotinib (PF2341066), and γ-secretase inhibitor (L685458). These results do not match the literature, which only reported *SLC43A1* cellular susceptibility to paclitaxel in lymphoblastoid B-cell lines (Njiaju et al., 2012). Therefore, the relationship between *SLC43A1* and drug sensitivity should be better investigated to validate its role as a new therapeutic target.

Among the highest dysregulated SLCs, *SLC44A4* was upregulated in 17 of 33 tumor types when compared to pooled GTEx control group, including the tumors of gastrointestinal origin. These findings are supported by previous studies, which reported that *SLC44A4* overexpression is associated with the onset of PRAD and PAAD (Mattie et al., 2016; Ma et al., 2019; McHugh et al., 2019). Similarly to *SLC34A2*, since *SLC44A4* was upregulated into immune-response (C2-C3) signatures, it could also play a key role in the activation of immunity. Although *SLC44A4* belongs to choline transporter-like family, it is associated to the transport and synthesis of acetylcholine, as well as the uptake of thiamine pyrophosphate (Song et al., 2013; Nabokina et al., 2014). Despite *SLC44A4* upregulation promotes the phospholipid synthesis, a link between *SLC44A4* expression and tumorigenesis has not been reported. In this field, further investigations should be undertaken to provide a deep knowledge of these SLCs involvement in such biological process.

The *SLC52A3* gene, better known as *C20orf54*, is a riboflavin transporter widely expressed in the intestine mediating the intestinal absorption (Yonezawa and Inui, 2013). Similarly, to *SLC44A4*, the upregulation of *SLC52A3* was observed in more than half of TCGA tumor types when compared to pooled GTEx control group. Despite the role of riboflavin in human health and cancer has been widely investigated in the last decades (Thakur et al., 2017), the involvement of its main transponder in tumorigenesis has been poorly investigated, except for few studies that focused on colorectal cancer and esophageal squamous cell carcinoma reporting *SLC52A3* up- and downregulation, respectively (Ainiwaer et al., 2013; Tutino et al., 2018; Li et al., 2022).

The *SLC24A5* and *SLC45A2* genes were found to be strongly associated with MAPKs inhibitors sensitivity. Specifically, our *in silico* analysis revealed that the overexpression of these SLCs genes increased the sensitivity of CCLE cells lines to PLX4720 (Anti-Braf) and AZD6244 (anti-MEK). As reported by César-Razquin and colleagues, this relationship could be due to the direct influx/efflux mediated by SLCs transporters or indirect effects, including the metabolic reprogramming of cancer cells (César-Razquin et al., 2018). Notably, *SLC24A5* and *SLC45A2* genes encode for melanosome-associated transporters both involved in melatonin synthesis in melanocytes and melanoma cells. Mechanistically, *SLC24A5* mediates the potassium-dependent sodium/calcium antiport influx and efflux involved in melanosome maturation (Rogasevskaia et al., 2019). On the other hand, the role of *SLC45A2* regulates the H^+^-dependent sucrose transport maintaining the melanosome pH balance and maturation and preserving tyrosinase (TYR) activity (Liu Y. et al., 2022). Although sequence variations in *SLC25A4* and *SLC45A2* have been described as associated with skin hypopigmentation and a higher risk for melanoma, their gene regulation mechanism is still unclear and should be further investigated (Reis et al., 2020).

Collectively, the gene expression analysis, performed comparing each tumor type with both matched normal tissue and pooled GTEx control group, highlights that the SLCs dysregulation strictly depends on their specific functions and it is generally tumor-specific. Since these genes play a key role in several tumoral features, such us metabolism and survival, deep remodulation of SLCs expression is mandatory during cancer development. Therefore, we focused on DNA methylation status, as major epigenetic effector of gene regulation, to better understand its involvement in the modulation of SLC genes.

Among the most significant SLCs, our DNA methylation analysis revealed that promoter hypomethylation of *SLC34A2*, *SLC44A4*, and *SLC52A3* was strongly associated with their overexpression in several tumors according to canonical mechanisms. Similarly, body region hypermethylation of *SLC44A4* and *SLC4A4* was positively correlated to gene expression, confirming the evidence that body region methylation status is a conserved marker of gene upregulation (Greenberg and Bourc’his, 2019). Previous studies highlighted the involvement of DNA methylation in the regulation of the *SLC44A4* and *SLC34A2* in colon adenocarcinoma, renal cell carcinoma, small-cell lung cancer, and papillary thyroid carcinoma (Ricketts et al., 2012; Xue et al., 2018; He et al., 2020; He et al., 2022). However, comprehensive analyses on methylation status of the promoter and body regions of these genes should be conducted in all tumor types to identify potential tumor-specific biomarkers. Unusual results were observed for *SLC26A10*, in which both promoter and body hypermethylation were related to gene overexpression in most tumor types. To the best of our knowledge, we described for the first time the positive correlation between promoter/body DNA methylation and expression of this gene in cancer providing a new point of view as a possible source for further investigations. Interestingly, OS and PFI analyses of cg06690548 probeset (*SLC7A11*) suggested that single-nucleotide resolution analysis of DNA methylation status may represent an independent prognostic factor in different tumors. Notably, the methylation status of this CG probeset was analyzed in several diseases, including fatty liver disease, hepatic steatosis, and Parkinson’s disease, however, no evidence was observed in tumors (Nano et al., 2017; Vallerga et al., 2020; Zhang X. et al., 2021). Therefore, this lacking data suggests that DNA methylation analyses should be extended to the SLCs mainly involved in tumorigenesis and cancer progression.

Overall, this *in silico* study provides an overview on the expression of SLC genes in the main tumor types focusing on their involvement in tumor growth, cancer metabolism, drug and immune response. However, it was not possible to achieve a comprehensive analysis of interplay between the most relevant SLCs due to the large number of analyzed SLCs, their multiple functions, as well as the high number of considered tumors. The obtained data suggested that the expression of several SLC genes is fine regulated to satisfy the molecules demand of cancer cells for metabolism and bimolecular synthesis. Since DNA methylation emerges as a key genetic regulation mechanism of these transporters, further studies should be addressed to investigate its regulatory role within tumor-specific context, as well as the SLCs dysregulation in cancer cells. In conclusion, our findings pave the way to a deeper understanding of the SLCs role in tumorigenesis to identify potential cancer-related biomarkers and targets for novel therapeutic strategies, including the selective targeting of SLCs and DNA methylation modulation.

## Data Availability

The datasets presented in this study can be found in online repositories. The names of the repository/repositories and accession number(s) can be found in the article/Supplementary Material. R codes and Raw data 1 and 2 were also deposited on Zenodo (https://doi.org/10.5281/zenodo.7974488).
