# Supplementary material for: In silico analysis of the solute carrier (SLC) family in cancer indicates a link among DNA methylation, metabolic adaptation, drug response, and immune reactivity
Source: Front Pharmacol. 2023 Jun 15;14:1191262. doi: 10.3389/fphar.2023.1191262 (PMC10308049; doi:10.3389/fphar.2023.1191262)
Supplement: Supplementary file 1 [file DataSheet1.docx]

Supplementary Material

In silico analysis of the Solute Carrier (SLC) family in cancer indicates a link among DNA methylation, metabolic adaptation, drug response, and immune reactivity

**Alessandro Lavoro, Luca Falzone, Barbara Tomasello, Giuseppe Nicolò Conti, Massimo Libra*, Saverio Candido**

*** Correspondence:** Massimo Libra: mlibra@unict.it

# Supplementary Figures and Tables

## Supplementary Figures


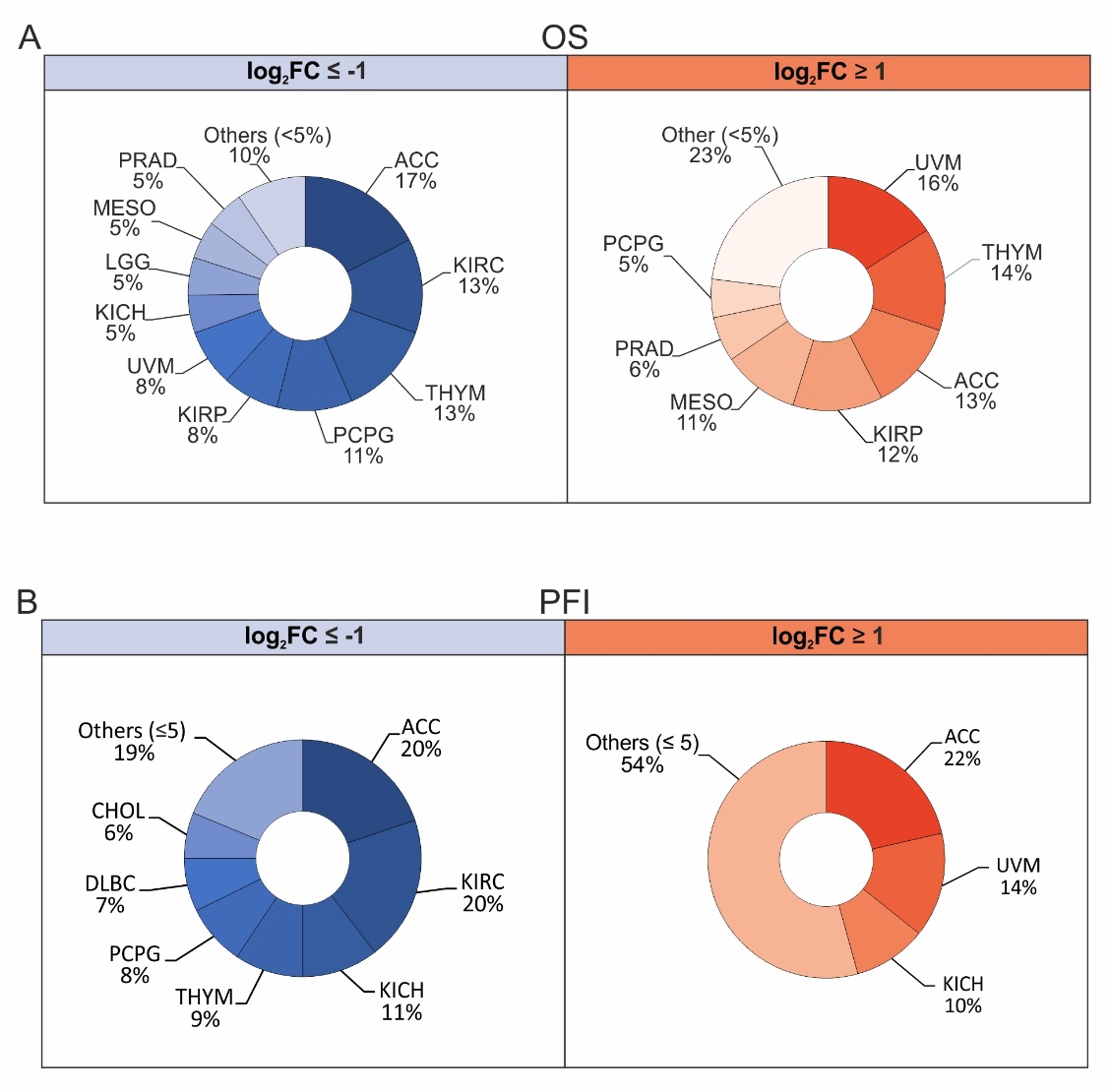


**Supplementary Figure 1.** Distribution of all **(A)** OS- and **(B)** PFI -associated DEGs among tumor types. The “Others” group includes all tumors that showed less than 5% of OS-associated DEGs.

**
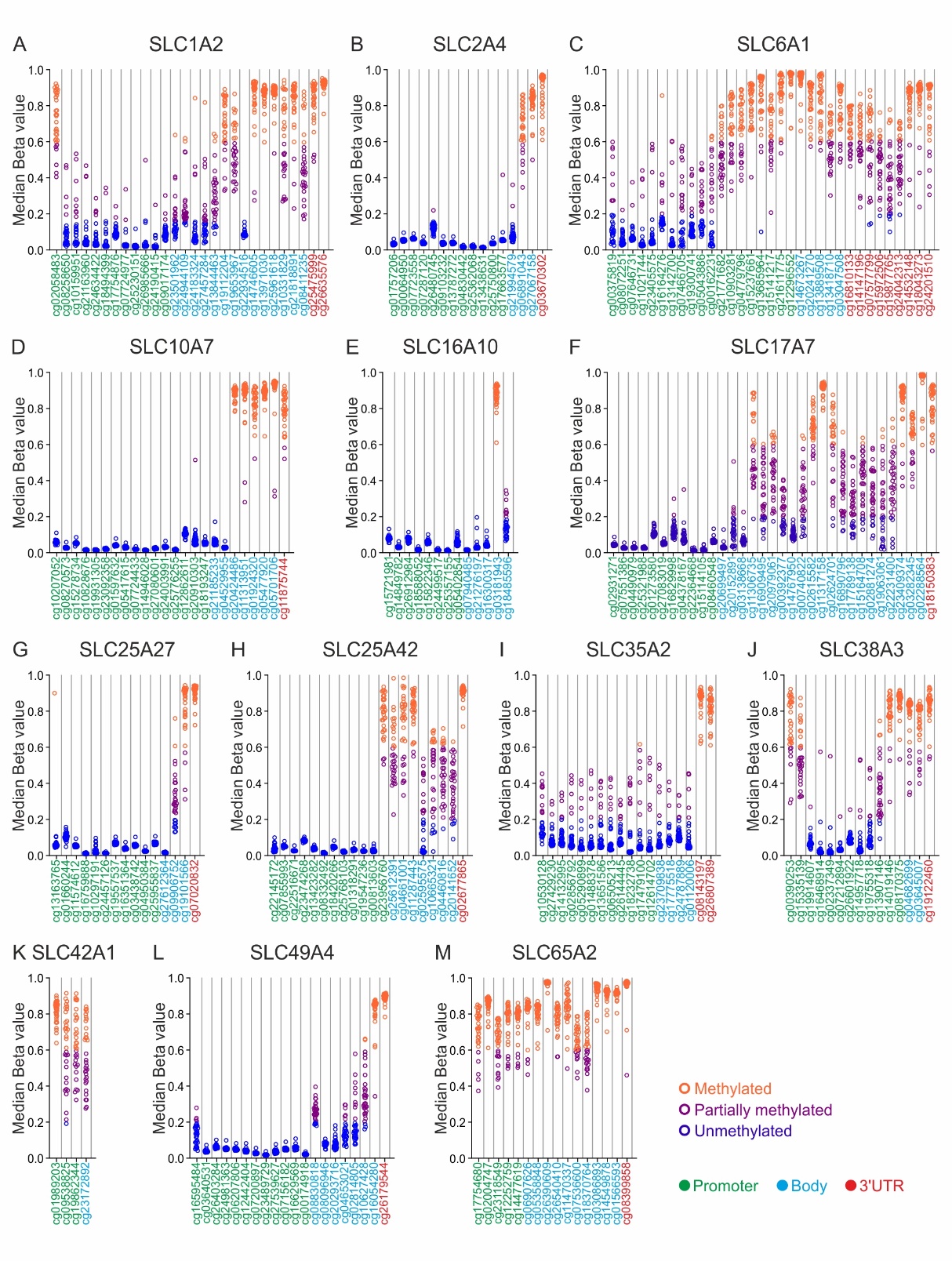
**

**Supplementary Figure 2.** DNA methylation status analyses of relevant SLCs for each TCGA Pan-cancer tumor type. **(A-M)** CG probesets median Beta values were computed for the relevant SLCs not included in gene expression and DNA methylation correlation analysis. Orange dots indicate the tumor types in which each CG probeset is methylated, purple dots indicate those showing partially methylated CG probesets, while blue dots indicate hypomethylation. The position of each CG probeset within the promoter, body, and 3’UTR region was indicated by green, cyan, and red labeling

**
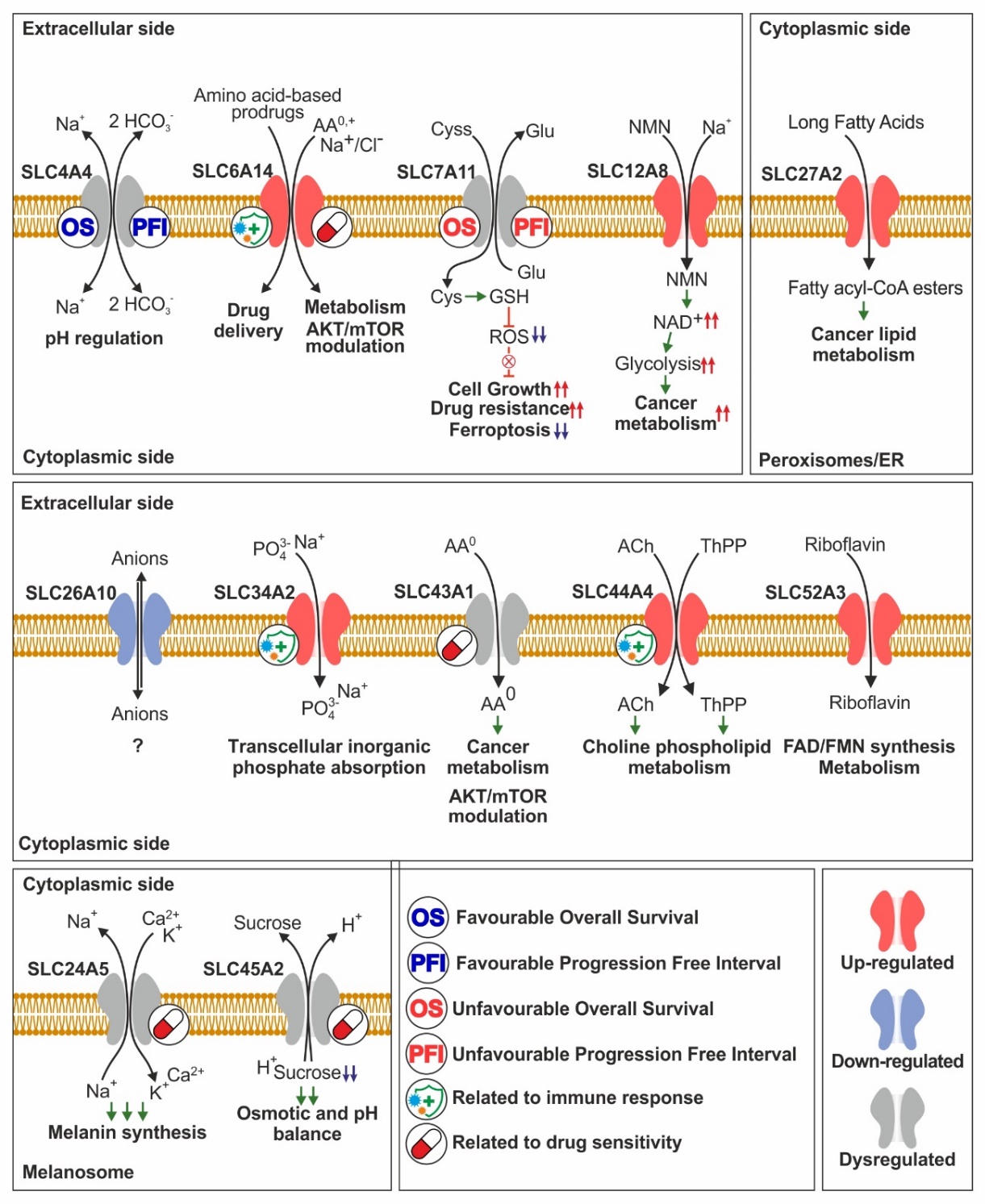
**

**Supplementary Figure 3.** Graphical representation of most relevant SLCs. The transmembrane transport mechanisms and the observed relationship with cancer are reported. Abbreviations: ACh, Acetylcholine; acyl-CoA, Acyl-coenzyme A; AA^0^, Neutral amino acids; AA^+^, Cationic amino acids; AKT/mTOR, AKT serine-threonine kinase/the mammalian target of rapamycin; Ca2^+^, Calcium ion; Cl^-^, Chloride ion; Cyss, Cystine; Cys, Cysteine; Glu, Glutamate; GSH, Glutathione; H^+^, Hydrogen ion; HCO3^-^, Hydrogen carbonate ion; K^+^, Potassium ion; Na^+^, Sodium ion; NAD^+^, Nicotinamide Adenine Dinucleotide; NMN, Nicotinamide Mononucleotide; PO43^-^, Phosphate ion; ROS, Reactive Oxygen Species; ThPP, Thiamine Pyrophosphate.

## List of Supplementary Tables:

**Supplementary Table 1.** SLC genes included in the analyses

**Supplementary Table 2.** Differential analysis of SLCs gene expression between TCGA cancer and pooled GTEx control group

**Supplementary Table 3.** Differential analysis of SLCs gene expression between each TCGA cancer and matched GTEx normal tissue

**Supplementary Table 4**. Differential analysis of SLCs gene expression between dead and alive at 5 years in TCGA Pan-cancer cohort

**Supplementary Table 5.** Differential analysis of SLCs gene expression between disease progression vs progression-free at 5 years in TCGA Pan-cancer cohort

**Supplementary Table 6.** Differential analysis of SLCs gene expression between C2-C3 (immune-response) and C4-C6 (immuno-quiet) in TCGA Pan-cancer cohort

**Supplementary Table 7.** Correlation analysis between SLCs gene expression and drug IC50 values tested on the CCLE cohort

**Supplementary Table 8.** Correlation analysis between SLCs gene expression and CG probesets DNA methylation values in Pan-cancer cohort

**Supplementary Table 9.** Median and range of DNA methylation values computed for each tumor type
